# Supplementary material for: DMRforPairs: identifying Differentially Methylated Regions between unique samples using array based methylation profiles
Source: BMC Bioinformatics. 2014 May 15;15:141. doi: 10.1186/1471-2105-15-141 (PMC4046028; doi:10.1186/1471-2105-15-141)
Supplement: Additional file 2 — DMRforPairs output for the comparison of A431-MCF7 and NA17018-NA17105. Please start from the HTML files in each folder. Available via the BMC Bioinformatics website. [file 1471-2105-15-141-S2.zip › 1394847754114233_MOESM2_ESM/A431_MCF7/figures/18235.pdf]

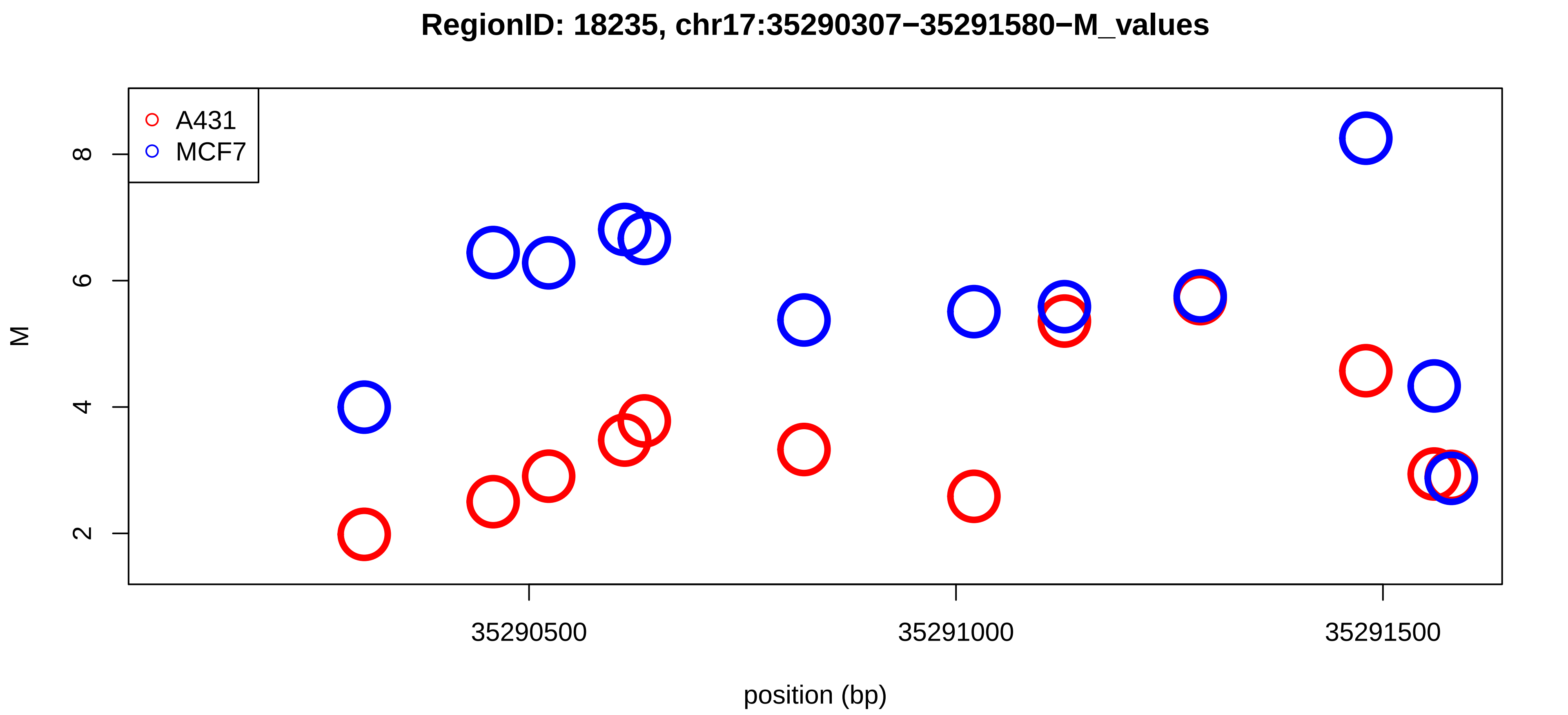
RegionID: 18235, chr17:35290307−35291580−M_values
A431
MCF7
M
2
4
6
8
35290500
35291000
35291500
position (bp)

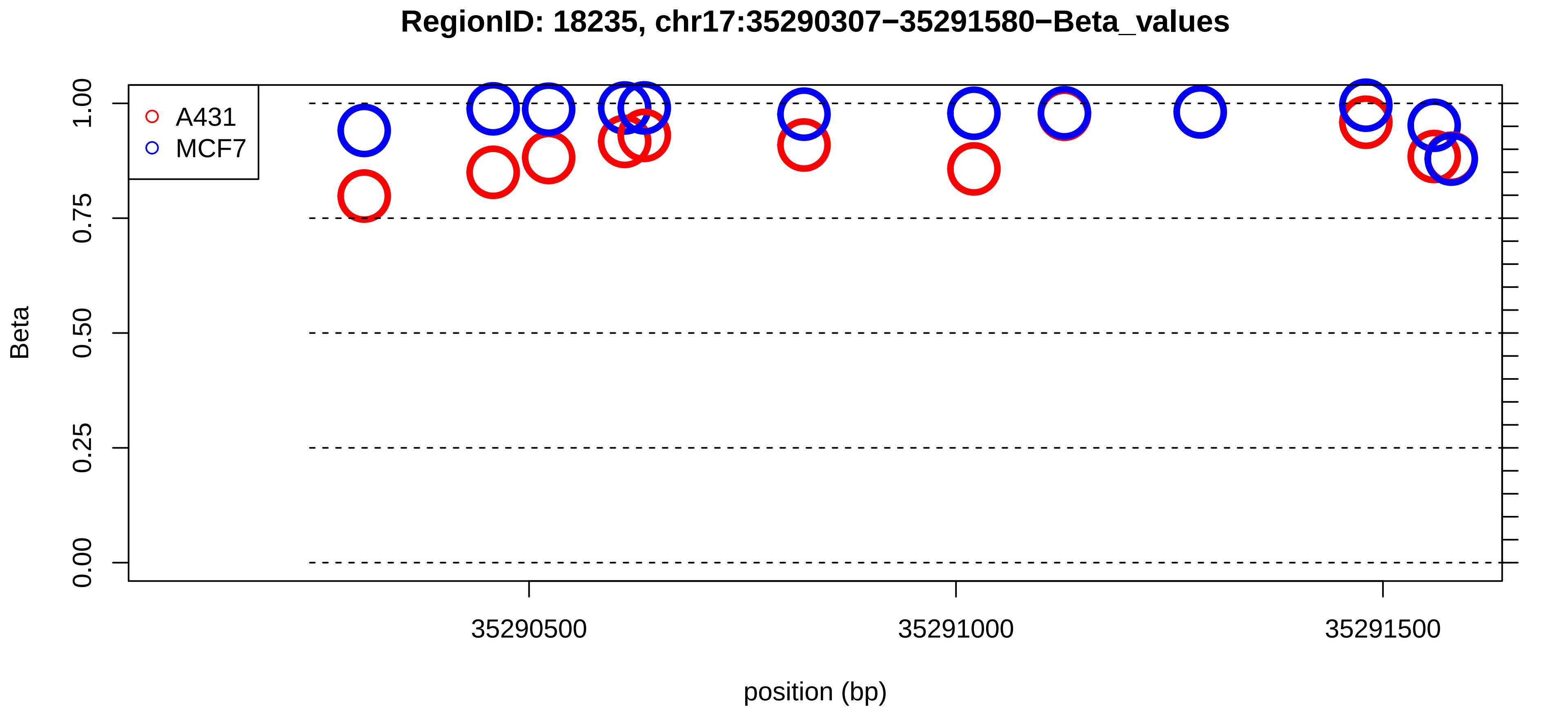
RegionID: 18235, chr17:35290307−35291580−Beta_values
A431
MCF7
Beta
0.00
0.25
0.50
0.75
1.00
35290500
35291000
35291500
position (bp)

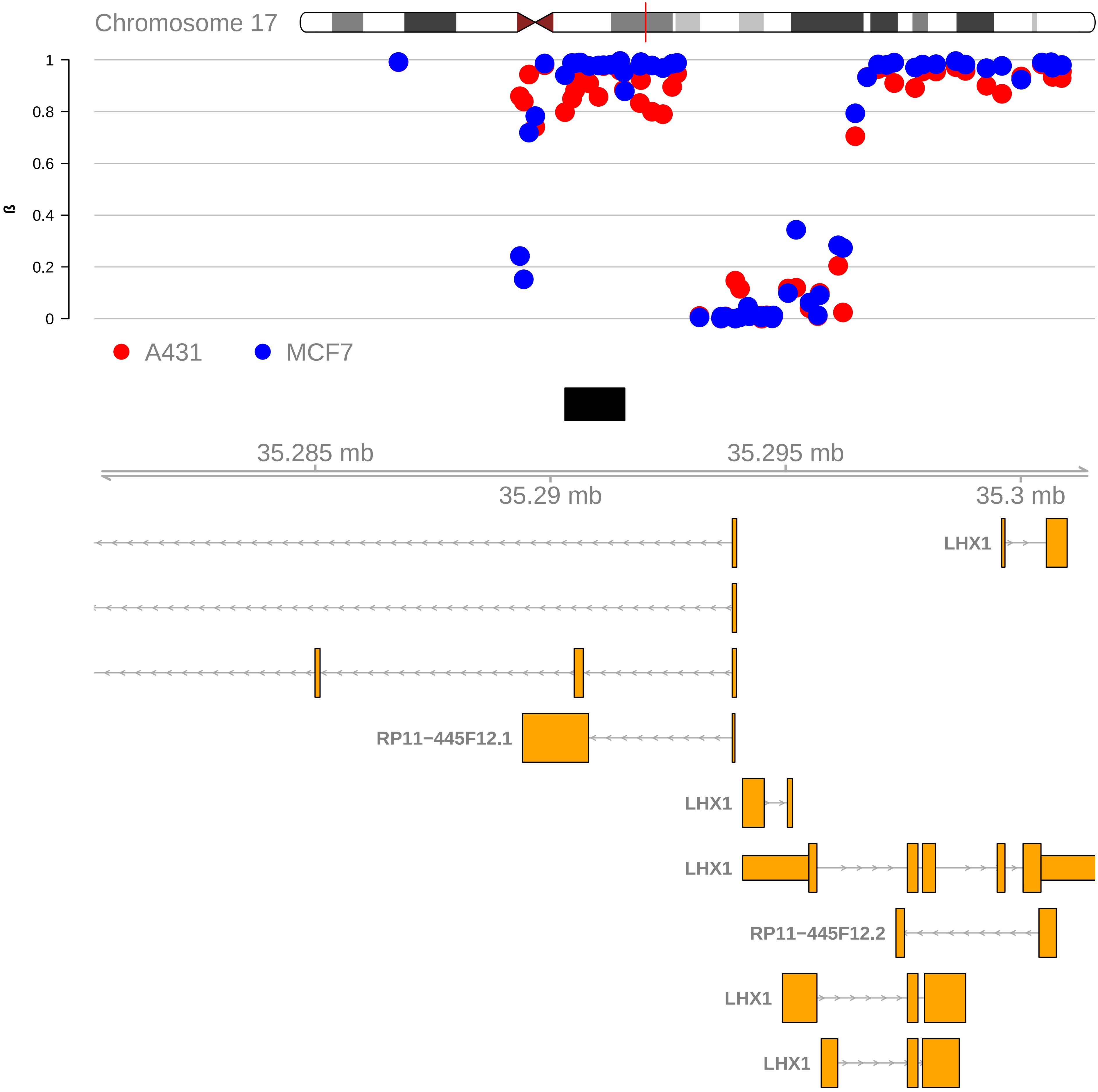

Chromosome 17
β
1
0.8
0.6
0.4
0.2
0
A431
MCF7
35.285 mb
35.295 mb
35.29 mb
35.3 mb
LHX1
RP11−445F12.1
LHX1
LHX1
RP11−445F12.2
LHX1
LHX1
